# Catalytic Fast Pyrolysis of Biomass Impregnated with Potassium Phosphate in a Hydrogen Atmosphere for the Production of Phenol and Activated Carbon

**DOI:** 10.3389/fchem.2018.00032

**Published:** 2018-02-21

**Authors:** Qiang Lu, Zhen-xi Zhang, Xin Wang, Hao-qiang Guo, Min-shu Cui, Yong-ping Yang

**Affiliations:** National Engineering Laboratory for Biomass Power Generation Equipment, North China Electric Power University, Beijing, China

**Keywords:** catalytic biomass pyrolysis, phenol, activated carbon, K_3_PO_4_, hydrogen atmosphere

## Abstract

A new technique was proposed to co-produce phenol and activated carbon (AC) from catalytic fast pyrolysis of biomass impregnated with K_3_PO_4_ in a hydrogen atmosphere, followed by activation of the pyrolytic solid residues. Lab-scale catalytic fast pyrolysis experiments were performed to quantitatively determine the pyrolytic product distribution, as well as to investigate the effects of several factors on the phenol production, including pyrolysis atmosphere, catalyst type, biomass type, catalytic pyrolysis temperature, and catalyst impregnation content. In addition, the pyrolytic solid residues were activated to prepare ACs with high specific surface areas. The results indicated that phenol could be obtained due to the synergistic effects of K_3_PO_4_ and hydrogen atmosphere, with the yield and selectivity reaching 5.3 wt% and 17.8% from catalytic fast pyrolysis of poplar wood with 8 wt% K_3_PO_4_ at 550°C in a hydrogen atmosphere. This technique was adaptable to different woody materials for phenol production. Moreover, gas product generated from the pyrolysis process was feasible to be recycled to provide the hydrogen atmosphere, instead of extra hydrogen supply. In addition, the pyrolytic solid residue was suitable for AC preparation, using CO_2_ activation method, the specific surface area was as high as 1,605 m^2^/g.

## Introduction

Pyrolysis is an important way for cost-effective utilization of lignocellulosic biomass materials (Bridgwater, 2012; Williams et al., 2016; Morgan et al., 2017). Among the various pyrolysis techniques, fast pyrolysis at medium temperatures around 500°C can convert solid biomass mainly into a liquid product known as bio-oil (Mohan et al., 2006; Wang et al., 2017). However, the poor selectivity in conventional pyrolysis process results in extremely complex composition of bio-oil, significantly lowering the grade of bio-oil for fuel or chemical applications (Kersten and Garcia-Perez, 2013; Carpenter et al., 2014). Therefore, catalytic pyrolysis technique was proposed to produce bio-oils with improved properties by using various catalysts, such as microporous zeolites, mesoporous catalysts, metal oxides, etc. Typically, zeolite catalysts possessed promising abilities of aromatization, alkylation and isomerization in biomass pyrolysis process for production of aromatic hydrocarbons due to their proper acidity and excellent shape-selectivity (Wang et al., 2014; Vichaphund et al., 2015). Compared with zeolites, mesoporous aluminosilicate catalysts were not effective to produce aromatic hydrocarbons, and they exhibit different performance based on various supports and modification metals. Generally, MCM-41 based catalysts had good capability on lowering the oxygen content of bio-oil, while use of MSU-S showed high selectivity of polycyclic aromatic hydrocarbons, heavy fractions and coke (Adam et al., 2005; Triantafyllidis et al., 2007). Metal modified SBA-15 could achieve cracking of heavy fractions (Lu et al., 2009). In addition, metal oxide catalysts such as MgO and TiO_2_ were also utilized for catalytic pyrolysis, from which the generated bio-oils were upgraded with lower oxygen contents (Stefanidis et al., 2011). In recent years, a more attractive technique, catalytic pyrolysis of biomass toward specific compounds through selective control of biomass pyrolysis process (known as selective pyrolysis) has gained extensive attentions, as it offers a promising way to facilitate the application of pyrolytic products (Theodore and Juan, 2013; Liu et al., 2014a; Shen et al., 2015; Zhang et al., 2017). Such catalytic/selective pyrolysis can be achieved via suitable biomass materials or proper catalysts under specific pyrolysis conditions, so as to obtain different valuable compounds, such as levoglucosenone (Lu et al., 2014; Zhang et al., 2015d; Ye et al., 2017), furfural (Lu et al., 2011; Zhang et al., 2014), 1-hydroxy-3,6-dioxabicyclo[3.2.1]octan-2-one (Fabbri et al., 2007; Zhang et al., 2015a), aromatic hydrocarbons (Vichaphund et al., 2015), 4-vinyl phenol (Qu et al., 2013), 4-ethyl phenol (Zhang et al., 2015c; Lu et al., 2016), 4-ethyl guaiacol (Lu et al., 2017), nicotyrine (Ye et al., 2016) and so on.

Lignocellulosic biomass basically consists of holocellulose and lignin. Lignin is a heterogeneous aromatic polymer formed by three basic monolignols with different C-O and C-C linkages (Akinosho et al., 2014). Pyrolysis of lignin follows complex mechanism to produce various phenolic compounds, and the product distribution is greatly affected by the biomass types and pyrolysis conditions (Zhang et al., 2012; Kalogiannis et al., 2015). Phenolic compounds are valuable chemicals that can be widely utilized in various industries. However, in conventional bio-oil, phenolic fraction is usually in a low concentration due to the limited lignin content in biomass and low monomeric phenolics yield during pyrolysis process. Moreover, phenolic fraction in bio-oil usually consists of many phenolic compounds without any dominant ones because of the poor pyrolysis selectivity. Currently, many studies have been conducted to achieve catalytic pyrolysis of biomass, focusing on either mixed phenolics or specific individual phenolic. Bu et al. (Bu et al., 2012, 2013) reported a way of microwave pyrolysis of Douglas fir sawdust with AC catalyst to obtain mixed phenolic compounds, and the phenolics concentration reached 75% (GC peak area%) in bio-oil. Lu et al. further found a new way to produce mixed phenolic compounds via catalytic fast pyrolysis of biomass impregnated with K_3_PO_4_ or mechanically mixed with solid base catalysts (Lu et al., 2013; Zhang et al., 2015b). Analytical pyrolysis chromatography-mass spectrometry (Py-GC/MS) experiments were conducted to determine the maximal phenolic concentration, which was as high as 68.8% (GC peak area%) from K_3_PO_4_ catalyst, or 68.5% (GC peak area%) from solid base catalyst. Besides the mixed phenolic compounds, specific individual phenolics are also the target products from pyrolysis of biomass, as single compounds are easier to be utilized than the mixed ones (Kim, 2015). *p*-Coumaric acid is a special component in certain herbaceous biomass materials like bagasse and bamboo, which is found to be the precursor of both 4-vinyl phenol and 4-ethyl phenol. Based on this fact, novel techniques were developed to produce these two single phenolic compounds, i.e, 4-vinyl phenol and 4-ethyl phenol from herbaceous biomass rich in *p*-coumaric acid under different pyrolytic conditions (Qu et al., 2013; Zhang et al., 2015c; Lu et al., 2016). Generally, 4-vinyl phenol could be produced from non-catalytic low-temperature fast pyrolysis process via direct decarboxylation of p-coumaric acid (Qu et al., 2013). Whereas, 4-ethyl phenol could be obtained from catalytic fast pyrolysis with Pd/SBA-15 catalyst or AC catalyst via decarboxylation and hydrogenation of p-coumaric acid (Zhang et al., 2015c; Lu et al., 2016). In addition, based on common woody biomass materials, a new way was proposed to prepare 4-ethyl guaiacol from catalytic fast pyrolysis of pine wood with Pd/SBA-15 catalyst (Lu et al., 2017). For an overview of the current studies, various pioneering research works have been done on the production of phenolics, but limited techniques have been successfully developed, especially on the individual phenolic compounds.

With previous studies on K_3_PO_4_ catalyzed pyrolysis of biomass for phenolics preparation and a brief exploitation on relevant mechanism (Lu et al., 2013; Zhang et al., 2015b), in this work, a new technique was proposed to prepare a monophenolic product, i.e., phenol. Phenol is the simplest phenolic compound and has a huge demand in the production of resins, fungicides, preservatives and drugs, etc. Previous studies have qualitatively confirmed the catalytic capability of K_3_PO_4_ on the formation of mixed phenolic compounds in inert atmosphere through analytical Py-GC/MS experiments (Lu et al., 2013; Zhang et al., 2015b). This study will display the promising catalytic capability of K_3_PO_4_ in the hydrogen atmosphere to obtain monophenol, rather than mixed phenolics. For better evaluating the capability of K_3_PO_4_, a lab-scale experimental setup was employed to quantitatively determine the distribution of pyrolytic products, which differed greatly from previous Py-GC/MS experiments. Experiments were carried out to investigate the effects of catalyst type (K_3_PO_4_, K_2_HPO_4_, and KH_2_PO_4_), pyrolysis atmosphere (nitrogen, hydrogen and mixed pyrolytic gas), catalytic pyrolysis temperature, catalyst impregnation content, and biomass type (hard wood, soft wood and herbaceous biomass) on pyrolytic product distribution, phenol yield and selectivity. Moreover, considering K_3_PO_4_ is also a chemical activation agent for AC preparation, pyrolytic solid residues contained K_3_PO_4_ were also subjected to activation process. In addition, the catalytic pyrolysis mechanism in hydrogen atmosphere was briefly explained.

## Experimental

### Materials

Three biomass materials were employed in this study, including poplar wood, pine wood and corn stalk. Poplar wood was used as the major feedstock for pyrolysis experiments. Prior to experiments, these biomass materials were crushed, then particles in size of 0.10–0.20 mm was sieved and dried at 105°C for 24 h, stored in a desiccator for pretreatment and experiments. The component and elemental composition results of these materials on the dry basis are given in the (Table S1).

### Pretreatment of biomass materials

Poplar wood and other biomass materials were pretreated to impregnate K_3_PO_4_ (or K_2_HPO_4_, KH_2_PO_4_) by using the incipient wetness impregnation method. The typical procedure was as follows. K_3_PO_4_ solutions in different concentrations were obtained using certain amounts of K_3_PO_4_ and 50 mL deionized water. Afterwards, poplar wood in certain amounts were soaked in these K_3_PO_4_ solutions, and treated with ultrasonic stirring for 12 h. Finally, the samples were dried at 105°C for 4 h and kept in a desiccator for experiments. Five pretreated poplar wood samples were prepared with different K_3_PO_4_ contents of 1, 5, 8, 10, and 15 wt%, respectively. K_2_HPO_4_ and KH_2_PO_4_ impregnated samples were prepared in the same way.

### Lab-scale fast pyrolysis experiments

**Figure 1:**
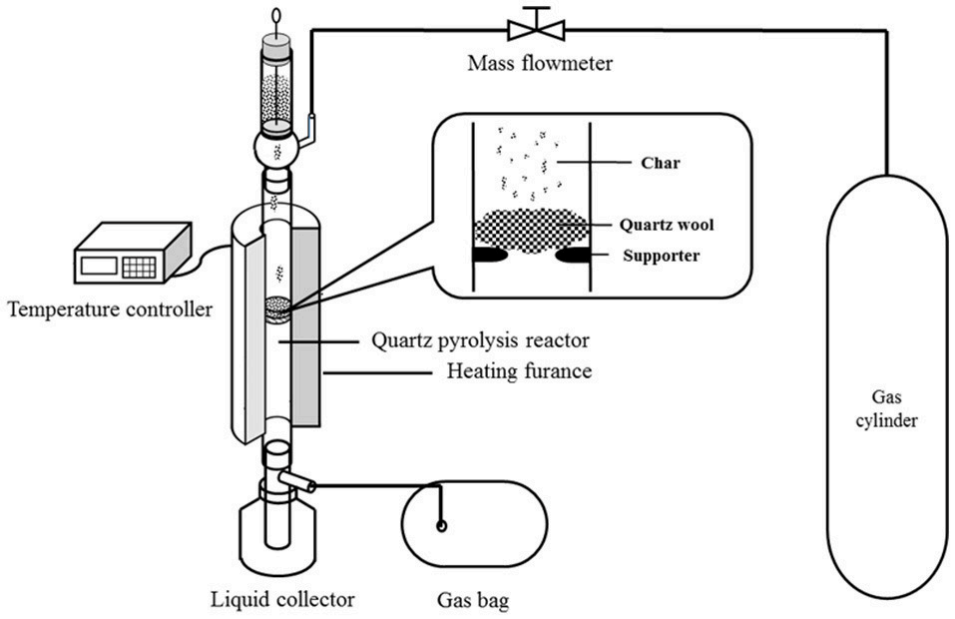
Lab-scale pyrolysis setup.

Catalytic pyrolysis experiments were conducted using a lab-scale pyrolysis setup, which is shown in Figure 1. The details of the experimental setup can be found in our previous studies (Lu et al., 2016; Ye et al., 2017). For each experiment, the amount of raw biomass was 3.00 g, while the quantity of the pretreated biomass varied to ensure the pure biomass quantity of 3.00 g. For example, 3.24 g biomass was used for the biomass impregnated with 8 wt% K_3_PO_4_. The feedstock was stored in the container, and then fed into the quartz pyrolysis reactor that was vertically placed in a heating furnace. Three different carrier gases were employed for experiments, i.e., nitrogen, hydrogen (6.00%, argon as shielding gas), and mixed gas (4.00% H_2_, 17.01% CO, 14.95% CO_2_, 6.06% CH_4_, helium as shielding gas), with the flow rate of 100 mL/min. The feeding of the feedstock lasted for 30 min with a strictly controlled rate, to ensure the temperature change in the reactor within ±3°C. The pyrolysis temperature was in the range of 400–700°C. A certain amount of quartz wool was placed in the quartz reactor to support the materials, preventing solid particles from falling into the condensation unit. The pyrolysis vapors were condensed in the condensation unit that was cooled by an ice and water mixture. The non-condensable gas was collected by a gasbag. In the end of the experiment, the pyrolysis quartz was cooled to room temperature within the carrier gas. The liquid and solid products were collected and weighed, and the quantity of the gas product was determined by difference.

### Analysis of pyrolytic liquid products

The liquid products from different pyrolysis experiments were not always homogeneous. Hence, after each experiment, a certain amount of ethanol was added into the condenser to mix with the pyrolytic liquid before taking out of the condenser, to obtain a homogeneous liquid for further analysis. The water content was determined by the Karl-Fisher method, and thus, the water content of the original pyrolytic liquid could be calculated. In addition, the chemical composition of the liquid product was analyzed by GC/MS (Perkin Elmer, Clarus 560). GC separation was accomplished using an Elite-35MS capillary column (30 m × 0.25 mm i.d., 0.25 μm film thickness) with a split ratio of 1:80. Helium (99.999%) was used as the carrier gas (1 mL/min). The GC oven was set at 40°C (stay for 2 min), then heated to 280°C with a heating rate of 15°C/min, followed by maintaining at 280°C for 2 min. The GC injector temperature was kept at 300°C. The detected products were determined based on NIST, Wiley Library and previous studies (Lu et al., 2013; Zhang et al., 2015b). Moreover, since phenol was the target product of this study, its actual yield was quantitatively determined via an external calibration method. Different amounts of pure phenol were injected into GC/MS to record the peak area values, so as to obtain a calibration curve in terms of phenol quantity and its chromatographic peak area. Based on this calibration line and the pyrolytic liquid yields, the actual yields of phenol under different pyrolysis conditions could be calculated.

### Preparation and characterization of ACs from pyrolytic solid products

The pyrolytic solid residues were the mixture of chars and K_3_PO_4_, which could be used to prepare ACs, because K_3_PO_4_ is a well-known chemical activation agent for AC preparation. Therefore, ACs were prepared via typical CO_2_ activation method. Thirty gram pyrolytic solid residue was heated from room temperature to 800^o^C with the heating rate of 20°C/min in nitrogen atmosphere with the flow rate of 500 mL/min, and then CO_2_ (500 mL/min) was used to replace N_2_ for activation at 800°C for 1 h, followed with cooling down to room temperature in nitrogen. The activated sample was washed at 60°C to neutral and dried in an oven for 24 h, to obtain the final AC. In addition, raw poplar wood and pretreated poplar wood with 8 wt% K_3_PO_4_ were also employed for preparation of ACs via CO_2_ activation with the same method.

The textural properties of the ACs were analyzed by the Autosorb-iQ-MP physisorption analyzer. The surface areas were determined using the Barrett–Emmett–Teller (BET) method. The pore volumes and pore size distribution were determined by the Barrett–Joyner–Halenda (BJH) method from the adsorption branch of the isotherms.

## Results and discussion

### Effects of pyrolysis atmosphere and catalysts on phenol production from poplar wood

#### Comparison of inert and hydrogen atmosphere

**Figure 2:**
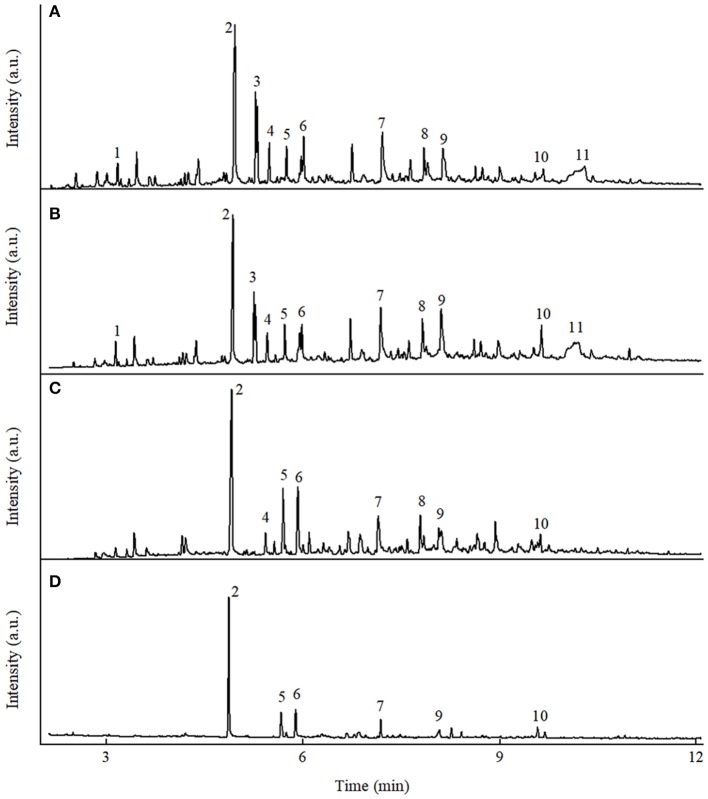
Typical ion chromatograms from GC/MS analysis of the pyrolytic liquid products from fast pyrolysis of poplar wood under different conditions. **(A)** Poplar wood in N_2_, **(B)** poplar wood in H_2_, **(C)** poplar wood with K_3_PO_4_ in N_2_, **(D)** poplar wood with K_3_PO_4_ in H_2_ [1: (acetyloxy)-acetic acid; 2: phenol; 3: 2,5-diethoxytetrahydro furan; 4: 2-hydroxy-3-methyl-2-cyclopenten-1-one; 5: 2-methyl phenol; 6: 4-methyl phenol; 7: 1,2-benzenediol; 8: 4-methyl-1,2-benzenediol; 9: 2-methoxy-4-vinyl phenol; 10: 4-ethylcatechol; 11: levoglucosan].

Fast pyrolysis experiments were firstly performed in nitrogen and hydrogen atmosphere at 550°C using raw and K_3_PO_4_-impregnated (8 wt%) poplar wood. The quantitative results of major pyrolytic product distribution are shown in Table 1. Typical ion chromatograms from GC/MS analysis of the pyrolytic products are shown in Figure 2. In addition, peak area% results for the major products under different pyrolysis conditions are presented in the (Table S2).

**Table 1:** Pyrolytic products distribution from poplar wood under different reaction conditions.

**Catalyst**	**Atmosphere**	**Char (wt%)**	**Liquid (wt%)**	**Gas (wt%)**	**Water content^a^ (wt%)**	**Phenol yield (wt%)**	**Phenol selectivity^b^ (%)**
–	N_2_	27.4	50.0	22.6	40.1	0.9 ± 0.0	3.0 ± 0.0
–	H_2_	24.5	53.8	21.7	38.2	2.1 ± 0.2	6.3 ± 0.6
K_3_PO_4_	N_2_	33.7	46.8	19.5	43.5	1.5 ± 0.1	5.7 ± 0.4
K_3_PO_4_	H_2_	30.8	50.9	18.3	41.4	5.3 ± 0.2	17.8 ± 0.7
K_2_HPO_4_	H_2_	33.7	46.6	19.7	44.3	3.8 ± 0.2	14.6 ± 0.8
KH_2_PO_4_	H_2_	31.6	47.1	21.3	43.9	3.5 ± 0.1	13.2 ± 0.3
K_3_PO_4_	mixed	30.4	48.1	21.5	40.3	5.0 ± 0.3	17.4 ± 1.0

a *Water content in the liquid product*.

b *Calculated by phenol yield divided by organic liquid yield*.

Non-catalytic fast pyrolysis of poplar wood in inert atmosphere has already been widely investigated in previous studies (Dong et al., 2012; Gu et al., 2013). As shown in Figure 2A, the pyrolytic liquid product contained various products without any dominant ones, which clearly indicated the poor selectivity in non-catalytic pyrolysis process in inert atmosphere. The yield of phenol was only 0.9 wt%, with the selectivity of only 3.0%, as shown in Table 1. When hydrogen atmosphere was employed in the non-catalytic process, remarkable changes were observed on the yields of pyrolytic products and the composition of the liquid product, as shown in Table 1 and Figure 2B. Such changes could be attributed to the fact that hydrogen participated the biomass pyrolysis reactions to help stabilize the intermediates and radicals, resulting in the decrease of char formation and the increase of liquid yield (Meesuk et al., 2011; Resende, 2016). In regard to phenol, its yield increased to 2.1 wt% in hydrogen atmosphere, with corresponding selectivity being 6.3%. Possible mechanism for the promotion of phenol formation in hydrogen atmosphere will be discussed in the following section. However, it is obvious that only hydrogen atmosphere was not sufficient to achieve phenol production, as the yield and selectivity of phenol from non-catalytic process were still very low.

Inspired by previous study that biomass impregnated with K_3_PO_4_ showed excellent performance on mixed phenolics preparation (Lu et al., 2013; Zhang et al., 2015b), catalytic fast pyrolysis of K_3_PO_4_-impregnated poplar wood was conducted in both inert and hydrogen atmosphere, with the results shown in Table 1. Significant changes in pyrolytic product distribution were identified as compared with non-catalytic process. According to Table 1, in both nitrogen and hydrogen atmosphere, the char yield increased, while the liquid and gas yields decreased in the presence of K_3_PO_4_, suggesting K_3_PO_4_ facilitated the polymerization and charring reactions of biomass toward char formation. Such catalytic effects were in accordance with the ability of K_3_PO_4_ that was efficient for AC preparation. Detailed catalytic mechanism of K_3_PO_4_ was remained to be explored, but previous studies have proposed some basic mechanistic descriptions. The potassium was considered to enhance the primary dehydration of substrate to form double bonds, which greatly accelerated the cross linking reactions under pyrolytic conditions to form char. Additionally, heating would also enhance the recombination reactions between neighboring heavy volatile fragments toward char (Zaror et al., 1985; Nik-Azar et al., 1997).

In regard to the pyrolytic liquid products in nitrogen atmosphere, holocellulose-derived products such as 2-hydroxy-3-methyl-2-cyclopenten-1-one, and levoglucosan were reduced greatly as shown in Figure 2C and Table S2. Phenol and other phenolic compounds became the major components in the liquid product, as the alkali catalysts could promote the decomposition of lignin (Kim, 2015). The above results were consistent with previous results that mixed phenolics were produced from K_3_PO_4_-catalyzed pyrolysis in inert atmosphere (Lu et al., 2013; Zhang et al., 2015b). However, K_3_PO_4_ only exhibited high selectivity on mixed phenolics, rather than monophenol. The yield of phenol was only 1.5 wt%, accompanied with the selectivity of only 5.7%. Whereas, when hydrogen was employed in the catalytic process, phenol was obtained with the yield of 5.3 wt% and the selectivity of 17.8%. The above results indicated that the synergistic effects of hydrogen atmosphere and K_3_PO_4_ were capable to achieve the phenol production.

#### Comparison of K_3_PO_4_, K_2_HPO_4_, and KH_2_PO_4_ catalysts

Besides K_3_PO_4_, two other potassium phosphates, i.e., K_2_HPO_4_ and KH_2_PO_4_, were also employed for catalytic fast pyrolysis of poplar wood in hydrogen atmosphere, to compare the catalytic capabilities of the three catalysts. The major pyrolytic product distribution results are given in Table 1. Typical ion chromatograms from GC/MS analysis of the pyrolytic liquids are given in the (Figure S1).

According to Figure 2, Figure S1, and Table 1, all the three catalysts exhibited the catalytic effects to produce phenol in hydrogen atmosphere. The phenol yields were 5.3, 3.8, and 3.5 wt% from K_3_PO_4_, K_2_HPO_4_, and KH_2_PO_4_, with the corresponding selectivity of 17.8, 14.6, and 13.2%, respectively. The results clearly indicated that the catalytic capability of the three catalysts was in the order of K_3_PO_4_ > K_2_HPO_4_ > KH_2_PO_4_. Due to the best catalytic capability of K_3_PO_4_ among the three catalysts, further experiments were all performed using K_3_PO_4_ in hydrogen atmosphere.

### Effects of pyrolysis temperature and K_3_PO_4_ contents on phenol production from poplar wood in hydrogen atmosphere

#### Effects of pyrolysis temperature

Temperature would significantly affect the biomass pyrolysis process and product distribution. Catalytic fast pyrolysis experiments were performed in the temperature range of 400–700°C using poplar wood with 8 wt% K_3_PO_4_ in hydrogen atmosphere. Table 2 gives the major pyrolytic product distribution results. Along with the increasing of pyrolysis temperature from 400 to 700°C, the char yield decreased continuously from 38.5 to 25.6 wt%, while the gas yield increased monotonically from 15.4 to 31.3 wt%. The liquid yield firstly increased and then decreased, and the maximal liquid yield was 50.9 wt% obtained at 550°C. Such changes agreed well with conventional effects of pyrolysis temperature on product distribution (Garcia-Perez et al., 2008; Kan et al., 2016). At pyrolysis temperature below 550°C, the increasing of pyrolysis temperature enhanced the pyrolysis reactions toward volatile products, to increase the liquid and gas products on the expense of char product. Whereas at temperature above 550°C, significant secondary cracking reactions took place to further promote the gas yield on the expense of char and liquid yields.

**Table 2:** Pyrolytic products distribution from catalytic fast pyrolysis of poplar wood with 8 wt% K_3_PO_4_ under different temperatures in hydrogen atmosphere.

**Temperature (°C)**	**Char(wt%)**	**Liquid(wt%)**	**Gas(wt%)**	**Water content^a^ (wt%)**	**Phenol yield (wt%)**	**Phenol selectivity^b^ (%)**
400	38.5	46.1	15.4	45.3	3.2 ± 0.2	12.7 ± 0.8
500	34.6	48.2	17.2	43.5	4.7 ± 0.1	17.3 ± 0.3
550	30.8	50.9	18.3	41.4	5.3 ± 0.3	17.8 ± 1.0
600	29.9	48.7	21.4	45.6	4.5 ± 0.2	17.0 ± 0.6
700	25.6	43.1	31.3	53.7	2.7 ± 0.1	13.5 ± 0.5

a *Water content in the liquid product*.

b *Calculated by phenol yield divided by organic liquid yield*.

In regard to the target product of phenol, both its yield and selectivity exhibited the same trend of first increasing and then decreasing along with the pyrolysis temperature. The maximal phenol yield was 5.3 wt% obtained at 550°C, together with the selectivity of 17.8%. During the pyrolysis process, decomposition of lignin firstly generated various phenolic compounds, further reactions of phenolics would occur to obtain phenol via elimination of substitutes by demethylation, demethoxylation, decarboxylation and others reactions (Zhang et al., 2012). Therefore, two processes would fundamentally affect the phenol yield during pyrolysis of biomass, i.e., the decomposition of lignin into phenolic compounds, and the substitute elimination of phenolics into phenol. Temperature was an essential factor for both processes. Below 550°C, increased temperatures would enhance the above reactions, while over 550°C, the secondary cracking and polymerization reactions would be the dominant ones to inhibit the above reactions. Such phenomena agreed with the change trend of liquid yield along with the increase of pyrolysis temperature.

#### Effects of K_3_PO_4_ content

Besides pyrolysis temperature, K_3_PO_4_ content in poplar wood was another important factor affecting the pyrolytic product distribution. Table 3 gives the major pyrolytic product distribution results from catalytic fast pyrolysis of poplar wood with different K_3_PO_4_ contents of 0, 1, 5, 8, 10, and 15 wt% at 550°C in hydrogen atmosphere. Along with the increasing of K_3_PO_4_ content, the liquid and gas yields decreased monotonically, while the char yield increased gradually, clearly reflecting the catalytic charring ability of K_3_PO_4_ to promote char formation. In regard to phenol, its maximal yield of 5.3 wt% was obtained from 8 wt% K_3_PO_4_, with the highest selectivity of 17.8% achieved under the identical condition. It is worth noting that when K_3_PO_4_ content was higher than 8 wt%, although the phenol yield decreased considerably, the phenol selectivity remained high. Possible reasons could be deduced from the two different catalytic effects of K_3_PO_4_ exhibited in the pyrolysis process. From one side, K_3_PO_4_ would promote the elimination of substitutes on phenolics to form phenol, resulting in a high phenol selectivity at elevated K_3_PO_4_ content. From the other side, K_3_PO_4_ would also catalyze the charring reactions to decrease the organic liquid product yield, especially at high K_3_PO_4_ content, which then decreased the formation of phenolics and further decreased the phenol yield.

**Table 3:** Pyrolytic products distribution from catalytic fast pyrolysis of poplar wood with different K_3_PO_4_ contents at 550°C in hydrogen atmosphere.

**K_3_PO_4_ content(wt%)**	**Char(wt%)**	**Liquid(wt%)**	**Gas(wt%)**	**Water content^a^ (wt%)**	**Phenol yield (wt%)**	**Phenol selectivity^b^ (%)**
0	24.5	53.8	21.7	38.2	2.1 ± 0.1	6.3 ± 0.2
1	27.3	53.2	19.5	38.8	3.1 ± 0.1	9.5 ± 0.2
5	28.1	52.6	19.3	39.3	4.5 ± 0.2	14.1 ± 0.6
8	30.8	50.9	18.3	41.4	5.3 ± 0.3	17.8 ± 1.0
10	34.6	47.3	18.1	44.9	4.5 ± 0.2	17.3 ± 0.8
15	40.2	43.7	16.1	49.9	3.7 ± 0.1	16.9 ± 0.3

a *Water content in the liquid product*.

b *Calculated by phenol yield divided by organic liquid yield*.

#### Adaptability of this technique on other biomass materials

In addition to poplar wood, the other two biomass materials (pine wood and corn stalk) were also employed for catalytic fast pyrolysis experiments, to determine the adaptability of this technique on different biomass materials. Poplar wood, pine wood and corn stalk represented typical hard wood, soft wood and herbaceous materials respectively. Table 4 gives the results from the three biomass materials with 8 wt% K_3_PO_4_ at 550°C in hydrogen atmosphere. Typical ion chromatograms from GC/MS analysis of pyrolytic liquids are given in the (Figure S2). As shown in Figure S2, pine wood presented the highest phenol yield and selectivity of 5.6 wt% and 18.1%, higher than those of poplar wood and corn stalk. Previous discussion has clearly stated that phenol was derived from lignin decomposition and phenolic substituents elimination. Based on Table S1, results of phenol yield and selectivity from different biomass materials strictly aligned with the lignin content in each material. Furthermore, since ash would catalyze the charring reactions to promote char formation (Yildiz et al., 2015), corn stalk had the lowest lignin content and highest ash content, and thus showed much lower phenol yield and selectivity. Based on the above results, it can be concluded that woody biomass materials were suitable to be used for phenol production via this technique.

**Table 4:** Pyrolytic products distribution from catalytic fast pyrolysis of different biomass materials with 8 wt% at 550°C in hydrogen atmosphere.

**Biomass**	**Char(wt%)**	**Liquid(wt%)**	**Gas(wt%)**	**Water content^a^(wt%)**	**Phenol yield (wt%)**	**Phenol selectivity^b^ (%)**
Pine wood	28.3	54.1	17.6	42.9	5.6 ± 0.2	18.1 ± 0.6
Corn stalk	28.5	51.0	20.5	41.8	2.5 ± 0.1	8.4 ± 0.3
Poplar wood	30.8	50.9	18.3	41.4	5.3 ± 0.3	17.8 ± 1.0

a *Water content in the liquid product*.

b *Calculated by phenol yield divided by organic liquid yield*.

#### Adaptability of simulated biomass pyrolytic gas for this technique

Hydrogen atmosphere was required in this technique for phenol production. However, due to the high cost of hydrogen, adoption of external hydrogen for phenol production was uneconomic. Fortunately, during biomass pyrolysis process, the non-condensable gas product mainly consisted of H_2_, CO, CO_2_, and CH_4_. Since the biomass pyrolytic gas has a certain amount of H_2_ (Ni et al., 2006), it is reasonable to predict that the biomass pyrolytic gas product might be recycled for the catalytic fast pyrolysis process. However, due to the limitation of the experimental setup, the pyrolytic gas product could not be recycled for experiments. Therefore, simulated pyrolytic gas, i.e., mixture of H_2_ (4.00%), CO (17.01%), CO_2_ (14.95%), CH_4_ (6.06%), and He, was employed for catalytic fast pyrolysis of poplar wood with 8 wt% K_3_PO_4_ at 550°C. The results are given in Table 1, and the typical ion chromatogram from GC/MS analysis of the pyrolytic liquid is given in the (Figure S3). According to Table 1, in the mixed gas atmosphere, the phenol yield and selectivity were similar to those obtained in the hydrogen atmosphere. Therefore, it can be concluded that the biomass pyrolytic gas was feasible to be recycled for catalytic fast pyrolysis process to produce phenol.

### Possible mechanism of phenol formation during K_3_PO_4_-catalyzed pyrolysis in hydrogen atmosphere

Based on the above results, possible mechanism for phenol production can be deduced. Due to the synergistic effects of K_3_PO_4_ and hydrogen, phenol was obtained in the catalytic fast pyrolysis process. Previous studies have analyzed the catalytic effect and mechanism of K_3_PO_4_ in inert atmosphere (Lu et al., 2013; Zhang et al., 2015b). Fast pyrolysis of lignin mainly follows the free radical mechanism, during which a large amount of radical species are produced from lignin decomposition (Demirbaş, 2000; Kim et al., 2014). Generally, these radical species can combine with H radicals to form stable phenolic compounds, also can undergo radical-coupling to form large molecule oligomers. Under non-catalytic conditions, very limited H radicals can be released from the formation of coke and oligomers. Therefore, due to the lack of H radicals, it is difficult for lignin-derived radicals to form phenolics. Whereas, the addition of K_3_PO_4_ significantly altered the product distribution. Hence it is reasonable to deduce that K_3_PO_4_ would catalyze the biomass pyrolysis process to generate hydrogen donors to stabilize the lignin-derived radicals (Lu et al., 2013; Zhang et al., 2015b). Previous works reported that holocellulose could act as hydrogen donors (Omori et al., 1998), and alkali metals had promoting effect on heterolytic cleavage reactions to help create more hydrogen radicals for stabilization of lignin-derived radicals (Julien et al., 1991; Yu et al., 2014). Therefore, K_3_PO_4_ could enhance the formation of hydrogen radicals during the pyrolysis process. As a result, yield of mixed phenolics was promoted, whereas the selectivity for phenol was still poor. Hence, it can be inferred that the presence of hydrogen would help to eliminate the substitutes of phenolics to obtain phenol as the predominant single product. In this study, discussion will be mainly concentrated on explaining the possible mechanism of substitute removal from phenolic compounds in the catalytic process in hydrogen atmosphere.

**Figure 3:**
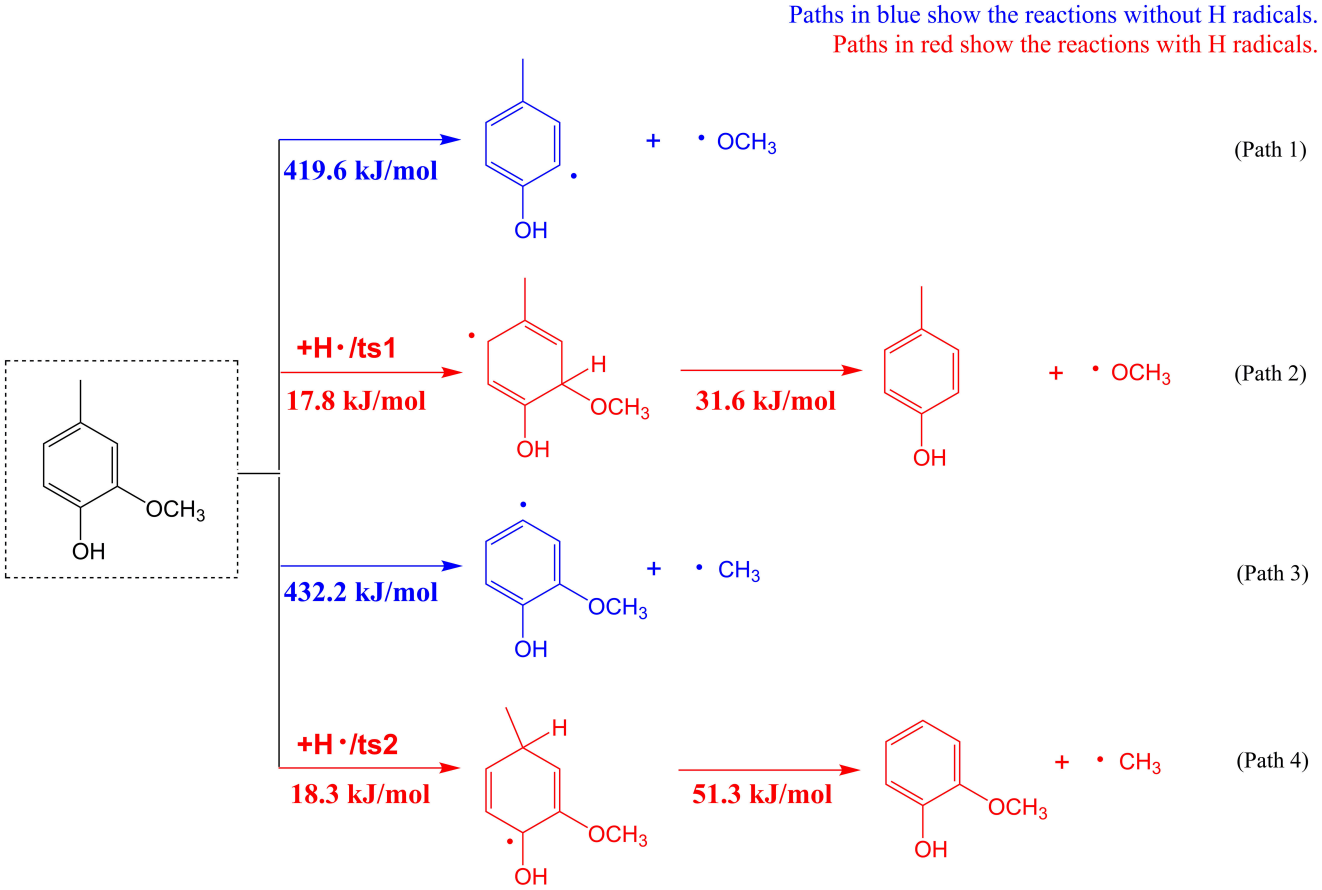
The elimination of substitutes on the model phenolic compound (2-methoxy-4-methyl-phenol) with or without H radicals.

Substitutes of phenolics are usually linked via C-O and C-C bonds to the aromatic ring of phenolics. Cleavage and removal of these substitutes are usually difficult and requires overcoming high energy barriers in inert atmosphere. Take 2-methoxy-4-methyl-phenol as a model phenolic compound, density functional theoretical (DFT) calculations based on Gaussian 09 software were performed to compare the substitute removal in the presence or absence of H radicals. Calculation details can be found in the Supplementary Material, and results of the pyrolytic pathways with or without H radicals are given in Figure 3. According to Figure 3, for removal of methoxyl group, the energy barrier of demethoxylation reaction without H radical is as high as 419.6 kJ/mol (Path 1), which means that this reaction is difficult to take place during the real pyrolysis process. Whereas, the energy barrier of demethoxylation reaction is significantly decreased to a very low value of 31.6 kJ/mol in the presence of H radical (Path 2), suggesting the reaction is very easy to occur. Similarly, removal of methyl group from 2-methoxy-4-methyl-phenol needs to overcome much lower energy barrier with H radical (Path 4) than without H radical (Path 3). The calculation results confirmed the viewpoint that the presence of H radicals would accelerate the removal of substitutes on the aromatic rings of phenolic compounds (Asmadi et al., 2011; Liu et al., 2014b). Therefore, combining above calculation results and previous studies, possible formation mechanism of phenol can be briefly explained. During the catalytic fast pyrolysis process, although K_3_PO_4_ was able to catalyze the decomposition reactions to generate hydrogen donors for stabilization of lignin-derived radicals (Lu et al., 2013; Zhang et al., 2015b), the radical amount was far from sufficient to eliminate the substitutes of phenolics to form phenol in the inert atmosphere. The utilization of hydrogen atmosphere in the catalytic process provided excessive hydrogen radicals that could be used to facilitate the elimination of substitutes on phenolics to form phenol. It should be stated that, as aromatic ring is stable, hydrogen radicals preferentially react with the substitutes of phenolics rather than aromatic rings, to produce phenol.

### Preparation and characterization of ACs from pyrolytic solid residues

Considering that pyrolytic solid residues contained K_3_PO_4_, conventional utilization as normal chars was infeasible before removing K_3_PO_4_. Whereas, K_3_PO_4_ is a good fertilizer for soil in agricultural application. But given the limitation of experimental conditions, test of pyrolytic solid residues for soil application cannot be achieved in this study. In addition, K_3_PO_4_ is also a well-known chemical activator for preparation of AC, which has already been applied in commercial scales, Therefore, activation experiments were further conducted by using the pyrolytic solid residue obtained from catalytic fast pyrolysis of poplar wood with 8 wt% K_3_PO_4_ at 550°C in hydrogen atmosphere, to confirm whether the solid residue was suitable for AC preparation. In addition, raw poplar wood and poplar wood impregnated with 8 wt% K_3_PO_4_ were also subjected to CO_2_ activation for AC preparation for comparison.

The textural properties of the ACs prepared from three different feedstocks are listed in Table 5. The specific surface area of the pyrolytic solid residue was only 36 m^2^/g. After CO_2_ activation, the specific surface area was as high as 1,605 m^2^/g, much higher than that of the AC from raw poplar wood (518 m^2^/g) and also a little higher than that of the AC from K_3_PO_4_-impregnated poplar wood (1,481 m^2^/g). Such results clearly indicated that pyrolytic solid residue was suitable for AC preparation, and agreed well with previous studies that activation of biomass with combined K_3_PO_4_ and CO_2_ would produce ACs with high surface area. The mechanism of the combined activation process has been clearly explained in previous studies (Laine and Calafat, 1991; Laine and Yunes, 1992). When heated in CO_2_ atmosphere, the interaction between potassium phosphate and the carbonaceous material would result in the formation of a potassium carbonate active center, while the remained free phosphate would compose a polymer net. The polyphosphates were supposed to be prone to form a “cover” which had good capability of keeping CO_2_ erosion from the surface of carbonaceous material to avoid excessive burn-off. Later, these phosphates were possible to locally interact with the activated carbon reactive sites, also might be along the edges of the twisted aromatic sheet linearly to form the slit-like micropores. In addition, this interaction allowed the aromatic sheet to be used for progressive CO_2_ attacks to form mesopores.

**Table 5:** Textural properties of ACs from different feedstocks by CO_2_ activation.

**Feedstock**	**BET surface area^a^ (m^2^/g)**	**Pore volume (cm^3^/g)**	**Average pore diameter (nm)**
Raw poplar wood	518	0.38	2.90
K_3_PO_4_-impregnated poplar wood	1,481	0.37	2.51
Pyrolytic solid residue	1,605	0.36	2.71

a *BET surface area of the pyrolytic solid residue was determined as 36 m^2^/g*.

## Conclusion

In this study, a new technique was proposed to produce phenol from catalytic fast pyrolysis of biomass impregnated with K_3_PO_4_ in hydrogen atmosphere, followed by co-production of AC from CO_2_ activation of pyrolytic solid residue. The results indicated that production of phenol would be greatly affected by the pyrolysis atmosphere, catalyst type, pyrolysis temperature and catalyst content. Based on the poplar wood, the maximum phenol yield of 5.3 wt% was obtained from catalytic fast pyrolysis of the poplar wood with 8 wt% K_3_PO_4_ at 550°C in hydrogen atmosphere, with corresponding selectivity of 17.8%. Pine wood was also suitable for phenol production, the phenol yield and selectivity from it were a little higher than those of poplar wood. Mixed pyrolysis gas was capable to provide the hydrogen atmosphere for phenol production, to avoid extra hydrogen supply. In addition, the pyrolytic solid residue was suitable to be precursor of AC, via CO_2_ activation method, the obtained AC owning specific surface area as high as 1,605 m^2^/g.

## Author contributions

ZZ, XW, and HG conducted the experiments. QL guided the design of experiments, analyzed the data and wrote the manuscript. MC revised the manuscript. YY coordinated the project. All authors reviewed, contributed to the revising, and agreed to the article's content.

### Conflict of interest statement

The authors declare that the research was conducted in the absence of any commercial or financial relationships that could be construed as a potential conflict of interest.
