# Supplementary material for: Catalytic Fast Pyrolysis of Biomass Impregnated with Potassium Phosphate in a Hydrogen Atmosphere for the Production of Phenol and Activated Carbon
Source: Front Chem. 2018 Feb 21;6:32. doi: 10.3389/fchem.2018.00032 (PMC5826322; doi:10.3389/fchem.2018.00032)
Supplement: Supplementary file 1 [file DataSheet1.docx]

Supplementary Material

**Catalytic fast pyrolysis of biomass impregnated with potassium phosphate in a** **hydrogen atmosphere for the production of phenol and activated carbon**

Qiang Lu*, Zhen-xi Zhang, Xin Wang, Hao-qiang Guo, Min-shu Cui, Yong-ping Yang

***Correspondence:** Qiang Lu: [qianglu@mail.ustc.edu.cn](mailto:qianglu@mail.ustc.edu.cn), qlu@ncepu.edu.cn

Table S1 Component and elemental composition of the three biomass materials on the dry basis (wt%).

|  | Poplar wood | Pine wood | Corn stalk |
| --- | --- | --- | --- |
| Component analysis |  | |  |
| Cellulose | 49.8 | 45.8 | 38.7 |
| Hemicellulose | 24.4 | 19.1 | 26.6 |
| Lignin | 23.3 | 30.7 | 17.9 |
| Extractive | 2.2 | 4.1 | 13.9 |
| Ash | 0.3 | 0.3 | 2.9 |
| Elemental analysis |  | |  |
| C | 49.6 | 50.9 | 45.4 |
| H | 6.3 | 6.5 | 6.0 |
| N | 0.1 | 0.1 | 1.5 |
| S | 0.1 | 0.1 | 0.2 |

Table S2 Peak area% results for major products from poplar wood pyrolysis under different catalytic conditions (%).

| Product | Poplar wood in N_2_  (Peak area %) | poplar wood in H_2_  (Peak area %) | poplar wood with K_3_PO_4_ in N_2_  (Peak area %) | poplar wood with K_3_PO_4_ in H_2_  (Peak area %) |
| --- | --- | --- | --- | --- |
| (acetyloxy)-acetic acid | 2.37 ± 0.03 | 2.18 ± 0.04 | 0.00 ± 0.00 | 0.00 ± 0.00 |
| phenol | 17.00 ± 0.16 | 23.08 ± 0.54 | 24.56 ± 0.54 | 39.23 ± 0.52 |
| 2,5-diethoxytetrahydro furan | 10.87 ± 0.17 | 8.18 ± 0.32 | 0.00 ± 0.00 | 0.00 ± 0.00 |
| 2-hydroxy-3-methyl-2-cyclopenten-1-one | 4.75 ± 0.06 | 4.01 ± 0.14 | 4.97 ± 0.10 | 0.00 ± 0.00 |
| 2-methyl phenol | 4.12 ± 0.11 | 4.65 ± 0.13 | 11.22 ± 0.21 | 11.09 ± 0.30 |
| 4-methyl phenol | 5.77 ± 0.21 | 4.60 ± 0.15 | 10.69 ± 0.27 | 12.29 ± 0.30 |
| 1,2-benzenediol | 5.39 ± 0.10 | 5.68 ± 0.21 | 6.67 ± 0.20 | 10.03 ± 0.25 |
| 4-methyl-1,2-benzenediol | 3.83 ± 0.06 | 4.94 ± 0.16 | 6.46 ± 0.20 | 0.00 ± 0.00 |
| 2-methoxy-4-vinyl phenol | 3.95 ± 0.21 | 4.78 ± 0.12 | 3.15 ± 0.07 | 2.63 ± 0.10 |
| 4-ethylcatechol | 1.34 ± 0.04 | 2.24 ± 0.05 | 1.91 ± 0.04 | 3.10 ± 0.07 |
| levoglucosan | 9.81 ± 0.29 | 7.27 ± 0.21 | 0.00 ± 0.00 | 0.00 ± 0.00 |





Figure S1 Typical ion chromatograms from GC/MS analysis of the pyrolytic liquid products from catalytic fast pyrolysis of poplar wood with different catalysts. **(A)** Poplar wood with K_2_HPO_4_, **(B)** poplar wood with KH_2_PO_4_, **(C)** poplar wood with K_3_PO_4_.





Figure S2 Typical ion chromatograms from GC/MS analysis of the pyrolytic liquid products from catalytic fast pyrolysis of different biomass materials. **(A)** Corn stalk, **(B)** pine wood, **(C)** poplar wood.





Figure S3 Typical ion chromatogram from GC/MS analysis of the pyrolytic liquid product from catalytic fast pyrolysis of poplar wood in the mixed gas atmosphere.

DFT calculation details

All cases were performed in Gaussian 09 software (Frisch et al., 2013). Equilibrium geometries of reactant, intermediates, transition states and products were optimized by using M06-2X method and the 6-31+G(d,p) basis set followed by frequency calculations. The reactant, intermediates and products had no imaginary frequency, while the transition states had only one imaginary frequency. Activation energies (reaction energy barriers) of reactions were estimated with the energy differences between transition states and reactant. But for homolytic cleavage reactions which have no transition states, bond dissociation energies are generally regarded as activation energies for comparison.

Reference

Frisch, M.J., Trucks, G.W., Schlegel, H.B., Schlegel, G.E., Robb, M.A., Cheeseman, J.R., et al. (2013). Gaussian 09, Revision D.01, Gaussian, Inc., Wallingford CT.
